# Bridging the Gaps in Thalassemia Care in Sri Lanka: Challenges and Way Forward

**DOI:** 10.1002/puh2.70122

**Published:** 2025-10-31

**Authors:** L. H. M. R. L. Ambillapitiya, L. S. M. Waidyasuriya, D. C. R. Weerakoon, R. M. P. M. Rathnayake, J. M. K. B. Jayasekara, H. D. W. T. Damayanthi, Thinley Dorji, Don Eliseo Lucero‐Prisno

**Affiliations:** ^1^ Department of Medical Laboratory Sciences Faculty of Allied Health Sciences General Sir John Kotelawala Defence University Werahera Sri Lanka; ^2^ Department of Pathology, Faculty of Medicine University of Peradeniya Kandy Sri Lanka; ^3^ Department of Nursing Faculty of Allied Health Sciences University of Peradeniya Kandy Sri Lanka; ^4^ Department of Internal Medicine Central Regional Referral Hospital Gelephu Bhutan; ^5^ Department of Global Health and Development London School of Hygiene and Tropical Medicine London UK

**Keywords:** health services accessibility, hemoglobinopathy, patient care, quality of life, social support, Sri Lanka

## Abstract

Thalassemia is the most common monogenic disease reported in Sri Lanka and has been a major health issue for decades. Although thalassemia major is preventable, there are about 60–80 births reported annually across the country. In Sri Lanka, the majority of them are managed at government hospitals, and healthcare facilities are provided free of cost, including blood transfusions and iron chelation therapies. Despite free treatments, patients have to bear certain expenses related to travelling, nutritional needs, and other necessities. Hemopoietic stem cell transplantation as a curative option is available only for a limited number of children in the government sector, whereas the cost is prohibitively high in the private sector. As consequence of this chronic disease, patients face persistent obstacles in education, employment, and social life. The lack of knowledge about the disease and its complications among the caregivers is a major challenge in the overall care cascade. This article highlights the measures that may be initiated by the thalassemia treatment centers in the state health sector of Sri Lanka to further improve the quality of life and living standards of patients. We recommend a national action plan to upgrade all treatment centers, enabling them to provide holistic care for thalassemia patients.

## 1 Introduction

Thalassemia, an autosomal recessive disease, is a common cause of anemia worldwide [1]. This chronic disease is an important health concern in Sri Lanka as it is the most prevalent monogenic disease reported in the country [2]. According to recent literature, an average of 80 babies with thalassemia major are born annually and nearly 2500 patients are currently undergoing treatments across districts in Sri Lanka [3]. β‐thalassemia major is the most common type, and Hb E/β‐thalassemia accounts for most of the rest [4]. In the recent years, the cost involved in providing services for thalassemia patients has been incurring increasing costs on the health budget of Sri Lanka [5].

**FIGURE 1:**
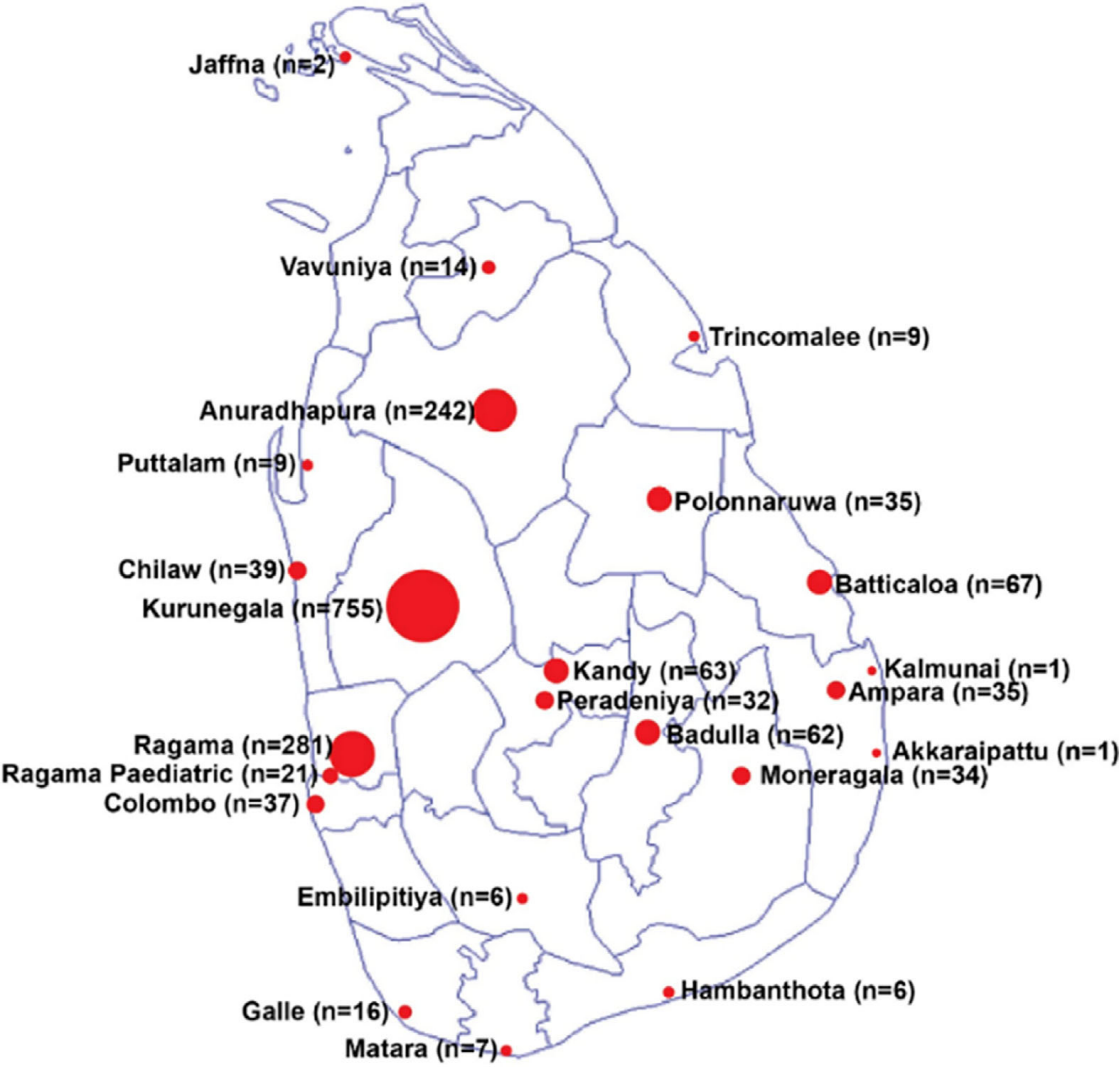
Number of patients with thalassemia registered at treatment centers of Sri Lanka [2].

The disease is highly prevalent in the Northwestern and North Central provinces [6], whereas the highest number of cases is reported from the Kurunegala district (Figure 1) [2]. The majority of patients are managed at state hospitals, and healthcare is provided free of charge, including blood transfusions and iron chelation therapies. Hemopoietic stem cell transplantation (HSCT) as a curative option is available only for a limited number of children of age less than 15 years in the government sector. It costs around 6–8 million Sri Lankan rupees ($20,000–$27,000) in the private sector, an amount that may not be affordable for the vast majority.  

**FIGURE 2:**
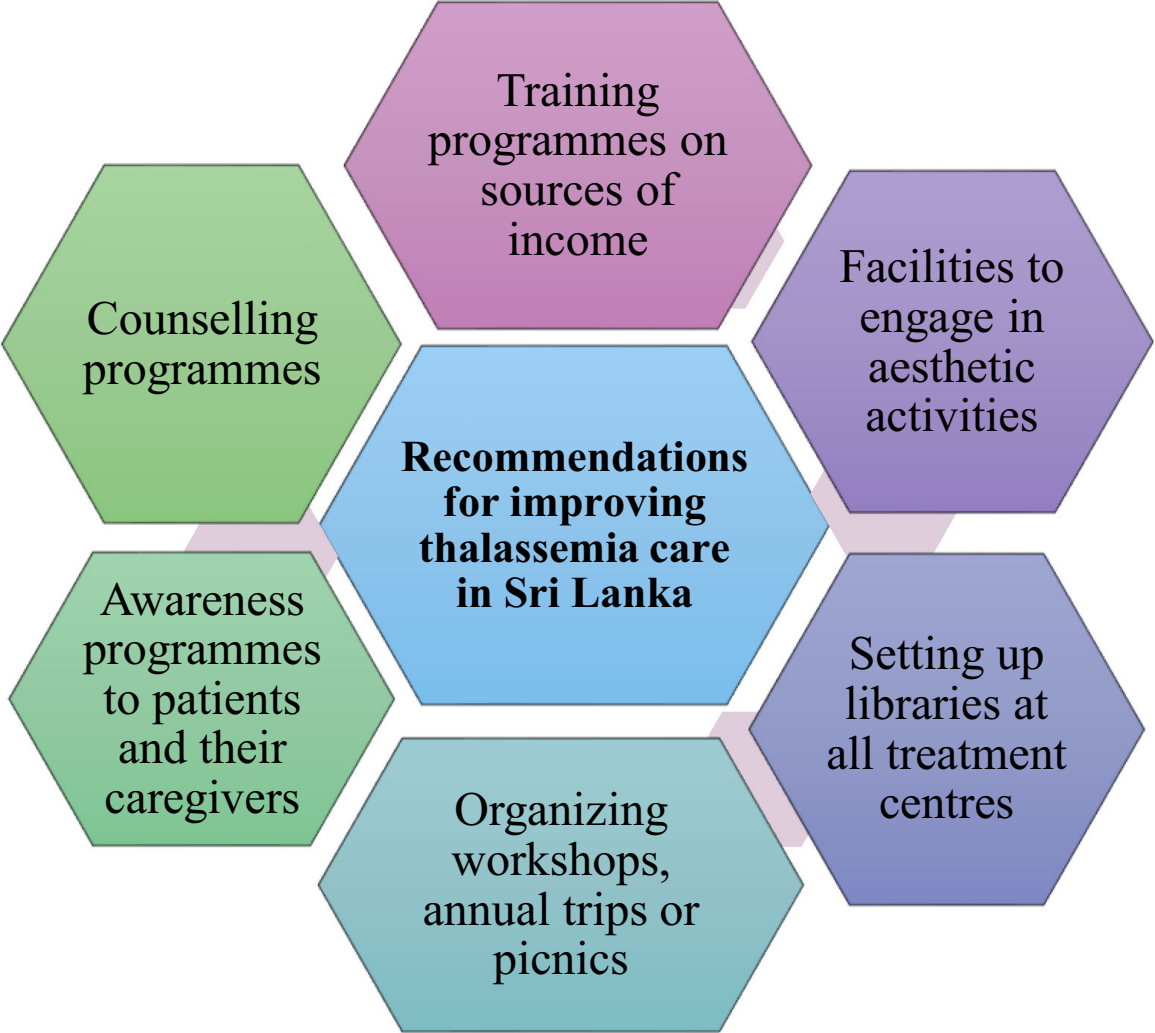
Recommendations for improving the living standards and quality of life of patients with thalassemia in Sri Lanka.

This chronic disease leads to various negative physical, mental, and social consequences [7]. Frequent hospital visits and blood transfusions have major impacts on day‐to‐day activities, including schooling, education, employment, and social life of transfusion‐dependent thalassemia (TDT) patients. Moreover, patients and their families encounter significant economic challenges due to out‐of‐pocket expenses, including costs for travelling and other essential needs associated with patient's care.

In addition to providing in‐hospital treatments by the thalassemia centers, there is a necessity of a holistic approach to address the nonclinical needs of these patients. Comprehensive support is needed for them to cope with the routine challenges. Empowering young patients with educational opportunities, skill development, employment support, and enriched social life experiences will significantly enhance their overall well‐being and enable them to lead a purposeful life. Collaborative efforts of medical practitioners and hospital administrators to address the nonmedical patient needs will even contribute to further improvements in life expectancy.

This article emphasizes key measures that can be adopted by treatment centers within Sri Lanka's state health sector to address broader patient needs. We recommend the implementation of a national‐level initiative focused on upgrading existing facilities and enabling the delivery of multidisciplinary, patient‐centered care across all centers of the country.

## 2 Challenges Encountered by Thalassemia Patients

### 2.1 Financial Burden

Thalassemia is a chronic disease that leads to a significant financial burden on families with affected children. Parents of such children must take them every 2–6 weeks to thalassemia treatment centers, which are located in main cities, like Kurunegala, Ragama, Anuradhapura, Badulla, Kandy, and Batticaloa. These frequent long‐distance travels are financially challenging, especially for families residing in rural and remote areas. Although the cost of blood transfusions and iron chelation therapies is borne by the government, the parents have to bear expenses for some medications, and other necessities of their children. The government sector has only a limited capacity for HSCT, whereas the cost of therapy in the private sector is prohibitively high. Consequently, many families are compelled to sell or mortgage their properties, even including the place they live. According to a recent case study conducted in the Kurunegala district among 75 thalassemia major patients registered at the National Thalassemia Centre, 47% of their parents were under debts due to overwhelming expenses for their children [8].

In most cases, financial instability is further exacerbated by the unemployment of parents or engagement in low‐income occupations like hired workers or daily wage laborers. A study in 2018 revealed that 59% of the families of 75 patients at the National Thalassemia Centre, Kurunegala had a monthly income that is less than 35,000 Sri Lankan Rupees ($120) [8]. Moreover, most mothers are unable to contribute to household income as they have to fully dedicate for the child's continuous care. The parents engaged in daily wage occupations suffer income loss on days spent accompanying their children to the hospital, and they face constant risk of job loss due to repeated absences. These financial challenges highlight the need for comprehensive social support systems and targeted interventions to minimize the burden on affected families.

### 2.2 Lack of Employment

In Sri Lanka, unemployment among thalassemia patients remains a significant socio‐economic concern. In developed countries like America, neither transfusion nor chelation is associated with the negative employment status. For example, in a study conducted in North America, the employment rate of adult thalassemia patients was comparable to that of the general American population [9]. The study, which was conducted around 2010, reported that 70% of adult patients had employments, with 67% engaged in full‐time occupations. This indicates that treatment sessions do not adversely impact employability in such settings.

In contrast, most of the adult thalassemia patients in Sri Lanka remain unemployed, lacking a consistent source of income and thereby being unable to financially support their families [8]. Both the government and private sectors show reluctance in recruiting individuals with TDT due to inconveniences caused by granting regular medical leaves. These emphasize the need for inclusive employment policies and workplace adjustments. In countries like Greece and Cyprus, 10% of employment positions in the public service are reserved for qualified thalassemia patients. In addition, special social benefits, such as travel allowances, transfusion leaves, municipal tax reductions, early retirement provisions, and disability pensions, have been granted [10].

### 2.3 Interrupted Education

The quality of life of school‐aged children living with thalassemia is significantly compromised by the chronic nature disease [11]. It is found that school functioning was the most impaired domain in thalassemic children [12]. Although a few patients with TDT have excelled in academics and qualified for university entrance, the majority experience educational disruptions caused mainly by missing schools, due to regular hospitalization, financial hardships, unstable family environments, and psychological distress. According to a case study conducted recently at the National Thalassemia Centre of Kurunegala using 75 thalassemia major patients, 57% of the patients in school age have given up schooling, and 70% have lost the opportunity of obtaining a continuous school education [8]. Conversely, a study involving Greek thalassemic patients revealed that 69% had completed their tertiary education [13].

Despite the presence of exceptional talents in art, music, dancing, and even in fields like information technology among these children, the burden of disease often hinders their ability of accomplishing special achievements through their talents. Therefore, educational support should be prioritized in the comprehensive care of children with thalassemia.

### 2.4 Hardships in Social Life

Social stigma associated with thalassemia significantly affects patients and their families, as they may experience prejudice or social isolation because of misconceptions about the disease. The under‐transfused patients commonly show gradual physical changes, such as skin discoloration, bone deformities, growth retardation, and impaired physical function. These progressive changes can diminish self‐esteem and self‐confidence, making social interactions difficult and often resulting in long‐term psychosocial consequences that extend into adulthood. Moreover, a significant proportion of patients choose to remain single, potentially due to the social exclusion associated with the disease [14]. In contrast, a study conducted in Greece indicated that only 34.5% of patients aged over 26 years remained single, suggesting more favorable social integration in some settings [13].

The social burden is not limited to the patient alone, and families also experience social embarrassment and may prefer to conceal the diagnosis from relatives and the community to avoid judgments [15]. Hence, it is essential to implement strategies that minimize the social burden and promote social acceptance of patients. Reduced morbidity through multidisciplinary care will help considerably in enhancing the chances of social achievements.

### 2.5 Lack of Awareness on Thalassemia

The lack of awareness on the disease, especially among patients’ family members, is a common challenge in the overall care cascade. Studies conducted in India have found that the knowledge of majority of the parents about their child's disease was not adequate [16]. In Sri Lanka, this issue is dominant in cases where children are raised by grandparents, often due to parental migration or abandonment. Proper awareness among patients and their caregivers about thalassemia and the importance of timely treatments is essential for effective disease management and minimizing complications.

## 3 Recommendations for Future Directions

Patients with severe thalassemia undergo multiple challenges in their daily lives, and all these are consequences of the disease. It is crucial to support and encourage patients in major aspects, like education, career, financial status, and social life, thereby motivating them to lead a hopeful life. Government‐funded treatment centers, along with the help of the Ministry of Health, healthcare professionals, and non‐governmental organizations (NGOs), can play a vital role by implementing feasible and effective measures to further improve their living standards and quality of life (Figure 2).

The Ministry of Health, in collaboration with the hospital administrators, may ensure that vocational training programs to both patients and their caregivers are provided at all treatment centers of the country. These programs can focus on making simple products, like shoes, bags, soap, food products, ornaments, home decors, jewelries, and other small accessories. Such small‐scale income sources are especially beneficial in settings where access to formal employment is limited. Moreover, with the involvement of the government, it is important to offer job opportunities to patients accompanied by supportive regulations such as flexible working hours or medical leave, particularly on treatment days.

Hospital authorities can assist the patients in education by setting up libraries and digital learning stations at the treatment units. Healthcare staff can encourage patients to engage in reading during treatment visits, whereas the support of hospital teachers or volunteers can promote ongoing education and enhance cognitive development. In addition, clinic hours and transfusion times may be adjusted to allow services out of school hours, enabling academic continuity of patients.

With the support of NGOs and volunteers, facilities to engage in music, arts and crafts, yoga sessions can be provided to promote mental relaxation in patients. Skills development workshops can be conducted by experienced personnel at all treatment centers, focusing on the development of social interactions, communication skills, personality, and self‐confidence of patients. Making them engaged in group activities will cultivate teamwork and leadership qualities, further aiding in navigating social challenges.

It is also essential to organize regular counseling sessions under the guidance of clinical psychologists, counselors, and mental health professionals aimed at improving the mental health of patients. Religious sessions can also be arranged with the assistance of clergy to provide emotional support and to foster a positive outlook on life.

The knowledge and the attitude of the family surrounding the patient are of utmost importance for ideal patient management. Therefore, awareness programs should be conducted regularly by medical professionals at all treatment centers, involving both patients and their caregivers to make them aware about the nature of the disease, the importance of timely blood transfusions and other treatments, possible cure options, and the significance of preventing thalassemia major births in their families. Furthermore, a national prevention policy with public awareness programs and population screening campaigns is vital for the overall disease eradication. The public health sector can actively collaborate with NGOs like “Lanka Thalassemia Circle” and “Thalassemia Foundation for Adult Patients” to strengthen community awareness and patient support systems.

In addition, certain measures implemented in other countries could be adapted to the local setup. For example, in India, the Rights of Persons with Disabilities Act adopted in December 2016 has recognized people with blood disorders, such as thalassemia as “persons with disabilities” [17]. The national policies of certain countries provide a range of social benefits for thalassemia patients, including transport allowances, municipal tax reductions, transfusion leaves, early retirement provisions, disability pensions, and reserved quotas in public sector employment and university admissions [10]. Out‐of‐pocket expenses for patients can be minimized through similar approaches.

In Sri Lanka, although some of these measures are already in practice, it is essential to expand their scope and standardize these practices across the country so that the entire thalassemia population is equally benefited.

## 4 Conclusion

Despite improved survival due to regular transfusion and iron chelation therapy, severe thalassemia patients often experience a diminished quality of life. Enhancing their living standards requires a holistic approach that addresses not only their medical needs but also educational, economic, psychological, and social dimensions. Social success, including educational achievements and interpersonal relationships, will also contribute in improving clinical outcomes. Effective multidisciplinary interventions should be backed by national policies and uniformly implemented across all treatment centers. Collaboration among key stakeholders, including government bodies, NGOs, and healthcare professionals, is essential to establish the delivery of sustainable and equitable care models that enable patients to achieve optimal well‐being and functional independence.

## Author Contributions

**L. H. M. R. L. Ambillapitiya**: conceptualization, methodology, investigation, writing – original draft, validation, visualization, writing – review and editing, formal analysis, resources, data curation. **L. S. M. Waidyasuriya**: conceptualization, investigation, validation, visualization, writing – original draft, methodology, formal analysis. **D. C. R. Weerakoon**: conceptualization, investigation, writing – original draft, methodology, validation, visualization, formal analysis. **R. M. P. M. Rathnayake**: conceptualization, investigation, writing – review and editing, methodology, validation, visualization, formal analysis, supervision, resources, project administration, data curation. **J. M. K. B. Jayasekara**: supervision, project administration, validation, writing – review and editing, methodology, conceptualization, formal analysis. **H. D. W. T. Damayanthi**: supervision, project administration, conceptualization, writing – review and editing, methodology, validation, formal analysis. **Thinley Dorji**: writing – review and editing, validation, formal analysis, supervision, project administration. **Don Eliseo Lucero‐Prisno**: writing – review and editing, validation, formal analysis, project administration, supervision.

## Conflicts of Interest

Thinley Dorji and Don Eliseo Lucero‐Prisno are editors of Public Health Challenges journal. They were blinded from the peer review process of this journal.

## Data Availability

Data sharing is not applicable to this article as no datasets were generated or analyzed during the current study.
